# The Effect of Skating Exercises as High-Intensity Interval Training on Elderly Stroke Patients

**DOI:** 10.3390/brainsci15070676

**Published:** 2025-06-24

**Authors:** Min-Su Kim

**Affiliations:** Department of Physical and Rehabilitation Medicine, Soonchunhyang University Cheonan Hospital, Cheonan 31151, Republic of Korea; 84730@schmc.ac.kr; Tel.: +82-41-570-2220; Fax: +82-41-570-2221

**Keywords:** cardiorespiratory fitness, exercise, high-intensity interval training, muscles, postural balance, rehabilitation, skating, stroke

## Abstract

Background/Objectives: High-intensity interval training (HIIT) can optimize recovery by complementing the low cardiovascular fitness intensities typically achieved in stroke rehabilitation programs. Skating exercise is an HIIT workout developed based on ice skating movements, and we investigated the effects of this exercise on the cardiorespiratory fitness of elderly patients with minor stroke. Methods: Participants aged 65 or older with a National Institutes of Health Stroke Scale score of 3 or lower were recruited. This study was designed as a randomized controlled trial, in which the intervention group engaged in skating exercises following HIIT, while the control group underwent moderate-intensity continuous training (MICT). Both groups of participants performed either HIIT or MICT for 20 min per day, four times a week, over three months. Results: A total of 34 elderly patients with minor stroke were recruited, with an average age of 70.7 years. For three months, no fall-down injuries or adverse cardiovascular or cerebrovascular events were reported among patients undergoing HIIT or MICT. Both the intervention and control groups showed significant increases in the measures of aerobic capacity after the intervention. However, the patients in the intervention group exhibited significantly greater improvements in peak oxygen uptake, ventilatory threshold, and peak MET (*p* = 0.005, *p* = 0.002, and *p* = 0.024, respectively). Additionally, the Berg Balance Scale (BBS) scores and the skeletal muscle mass index showed significantly greater enhancements in the intervention group compared to the control group (*p* = 0.032 and *p* = 0.032). Conclusions: In conclusion, skating exercise could be a safe and effective HIIT protocol for elderly people who have experienced a minor stroke.

## 1. Introduction

Minor strokes are generally defined as strokes with a National Institutes of Health Stroke Scale (NIHSS) score of 3 or less, accounting for approximately 35% of all stroke occurrences [1,2]. Stroke specialists have focused on the rehabilitation and recurrence prevention of moderate to severe stroke patients who exhibit significant muscular weakness and cognitive impairments, while minor stroke patients have not received much attention [3]. However, it has been reported that 28% of minor stroke patients are unable to live independently [4]. Additionally, the incidence of cognitive impairment requiring reliance on others is known to be 25%, and the likelihood of stroke recurrence is reported to be higher than that of the general population [5]. To prevent the recurrence of strokes in minor stroke patients, antithrombotic medications and drugs for managing risk factors such as hypertension, diabetes, and hyperlipidemia are widely prescribed [6]. Nevertheless, fundamentally enhancing functional independence and improving cardiorespiratory fitness (CRF) to reduce the risk factors for ischemic strokes and atherosclerosis (e.g., hypertension, obesity, low CRF) are crucial for stroke patients [7,8].

Cardiorespiratory fitness refers to the ability of the circulatory and respiratory systems to efficiently supply oxygen to the muscles during sustained physical activity [9]. It is a key indicator of overall health and well-being, and higher levels of CRF are associated with a reduced risk of various diseases and increased longevity [9]. Aerobic exercise methods can be broadly categorized into high-intensity interval training (HIIT) and moderate-intensity continuous training (MICT), utilizing various exercise techniques such as walking, running, cycling, and swimming [10]. Stroke patients often experience muscle weakness, hemiplegia, balance dysfunction, and cognitive impairments, which is why the MICT protocol utilizing bicycles or treadmills has primarily been implemented [11,12].

Recently, there has been active research aimed at applying HIIT in rehabilitation programs to more efficiently enhance CRF in stroke patients. Several previous studies have reported that stroke patients who underwent HIIT exhibited greater improvements in CRF, quality of life, and independence in daily activities compared to those who underwent MICT [13,14,15]. However, HIIT is difficult to implement in stroke patients with moderate to severe neurological impairments who experience muscle paralysis and balance dysfunction [16]. Even in minor stroke patients, the target group has primarily been younger elderly individuals [14,15,17,18]. The majority of strokes occur in the elderly population, with 75% of strokes occurring in individuals aged 65 and older [19]. Elderly individuals often struggle to self-manage the intensity (speed and height) of exercise, and many fear engaging in HIIT on a treadmill due to the risk of falling and sustaining serious injuries [16]. Additionally, due to decreased muscle strength, they face challenges in performing adequately intense cycling exercises [20].

In this context, skating exercise can serve as an appropriate HIIT protocol for patients who have experienced minor strokes at an advanced age. Studies conducted on healthy individuals have reported that skating exercise can enhance CRF, balance functions, and strengthen the quadriceps muscles [21,22,23]. Additionally, it has been reported that skating exercise improves CRF and quality of life in younger elderly populations who are minor stroke survivors [24]. However, research conducted on the elderly population over the age of 65, which constitutes the majority of actual stroke patients, is limited. Therefore, the purpose of this study is to investigate whether applying skating exercises, an HIIT protocol, can safely improve CRF, balance function, and muscle strength in elderly minor stroke survivors and to compare the effects with MICT.

## 2. Materials and Methods

### 2.1. Participants

Patients aged between 65 and 75 years with minor stroke, presenting a National Institute of Health Stroke Scale (NIHSS) score of 3 or lower at the time of onset, were recruited from the outpatient clinic of the tertiary hospital. All subjects were individuals with a first-ever stroke onset and were registered in the study within three months of onset. Every participant was diagnosed with a stroke through examination by a neurologist and brain magnetic resonance imaging (MRI). Exclusion criteria were based on existing studies and are as follows: (1) patients who are unable to understand the process of skating exercises or the evaluation of outcome measures due to cognitive dysfunction (score of <20 on the Korean version of the Mental State Exam) or aphasia (failure to follow two instructions) and (2) patients with major cardiovascular, psychiatric, or musculoskeletal disorders, such as myocardial infarction, schizophrenia, acute bone injury, etc. [25]. All caregivers and participants provided written consent to provide information, and they could withdraw from the study at any time.

### 2.2. Study Design

This study was designed as a single-blind, randomized controlled trial. The participants were randomly assigned to two groups. The intervention group performed skating exercises, while the control group engaged in aerobic treadmill exercises. After the baseline assessment, the participants were randomized using web-based software (Research Randomizer 4.0, http://www.randomizer.org, accessed on 1 July 2021) according to a 1:1 allocation with variable block sizes and site-specific stratification. Researchers not involved in recruitment, consent, or data collection generated the randomization order and maintained its confidentiality until the completion of the baseline assessment to ensure concealed allocation. The patient was aware of whether they were doing HIIT or MICE. However, the evaluator conducted tests on the patient’s cardiovascular fitness and other assessments without knowing which group the patient belonged to. One evaluator assessed all participants.

### 2.3. HIIT Protocol

The HIIT protocol implemented by the intervention group utilized skating exercises [24]. The participants alternated between high-intensity and low-intensity exercises, each lasting one minute. The total duration of the HIIT skating exercise program was 20 min, and this exercise protocol was conducted by physical therapists experienced in stroke rehabilitation and specialists in rehabilitation medicine. The high-intensity exercise aimed for 80% of maximal heart rate reserve (HRR) while maintaining a Borg rating of perceived exertion (RPE) of 13–15 [14,24]. Low-intensity exercise was performed targeting 50% of HRR (RPE 9–11) [14,24].

**Figure 1:**
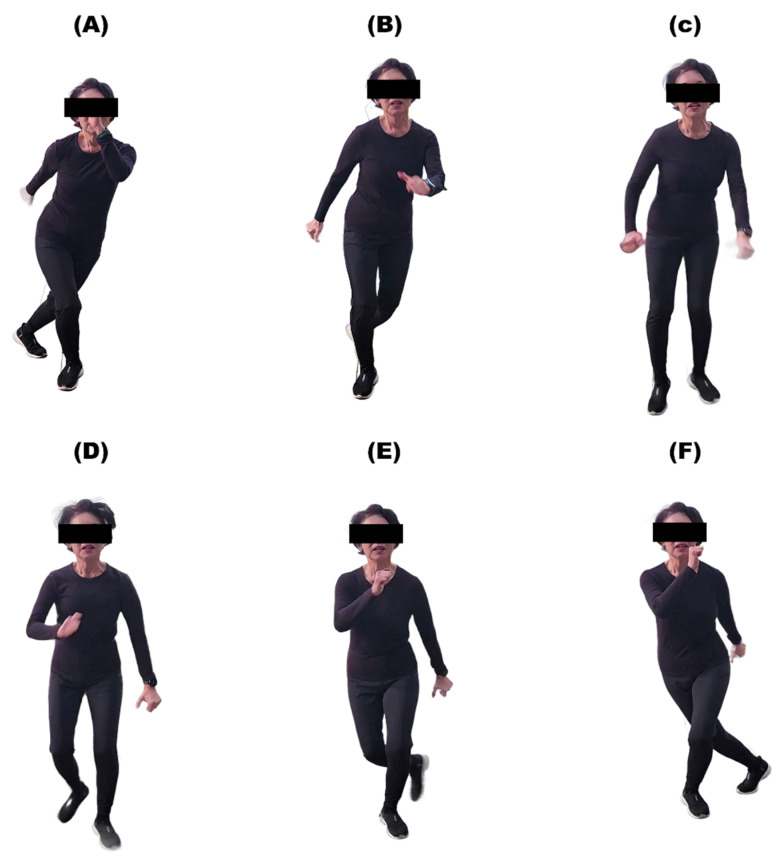
Continuous movement of skating exercise. (**A**) The patient stands on one leg with the knee slightly bent, shifting the center of gravity downward, while the other foot is positioned behind the body. (**B**–**E**) The patient jumps sideways to the left and lands on the left leg. The right leg is brought behind the left ankle. Simultaneously, the arms and legs are alternated, similar to speed skating. (**F**) The patient jumps to the right with the right leg to change direction. This completes one repetition.

The detailed stages of the skating exercise are as follows (Figure 1): First, the patient stands on one leg while holding the other leg behind their body after warming up. At the same time, the patient bends their knee slightly to shift their center of gravity downward. This position is similar to a small squat. Second, the patient jumps sideways to the left and lands on the left leg. They bring the right leg behind the left ankle. Simultaneously, they alternate their arms and legs in a manner similar to speed skating. Third, the patient jumps to the right with the right leg to change direction. This completes one repetition.

The patient is able to freely adjust the speed of exercise, the angles of the knees and ankles, and the degree of quadriceps usage according to their condition and functional capabilities while performing skating exercises.

### 2.4. MICT Protocol

The MICT protocol for the control group utilized a treadmill-based aerobic exercise program that is commonly employed for stroke rehabilitation [26,27]. The MICT protocol adjusted the treadmill’s incline and speed to maintain 40–50% HRR (RPE, 9–11), and exercise was performed continuously for 20 min. Patients from both groups engaged in a 15-min full-body stretching routine to prevent injuries before commencing exercise.

Patients from both the intervention and control groups participated in their respective exercise programs four times a week for a duration of three months. If a patient wished to stop exercising or exhibited symptoms such as dizziness or difficulty breathing, the exercise was immediately halted.

### 2.5. Outcome Measures

The primary outcome measure was CRF indicators. The participants’ CRF was assessed through cardiorespiratory exercise testing using the modified Bruce protocol [28]. The CRF measure was divided into aerobic capacity, cardiovascular response, and ventilatory response. Aerobic capacity was determined by the peak oxygen consumption (VO_2__peak_), ventilatory threshold (VT), and peak metabolic equivalents (MET_peak_).

Cardiovascular responses were assessed based on resting systolic blood pressure (SBP_rest_), resting diastolic blood pressure (DBP_rest_), peak systolic blood pressure (SBP_peak_), peak diastolic blood pressure (DBP_peak_), resting heart rate (HR_rest_), peak heart rate (HR_peak_), and peak oxygen pulse (O_2_pulse_peak_). Ventilatory responses were evaluated based on peak minute ventilation (VE_peak_), peak tidal volume (Vt_peak_), peak minute ventilation/carbon dioxide consumption (VE/VCO_2peak_), and peak respiratory rate (RR_peak_).

The secondary outcome measures included the assessment of balance function and skeletal muscle mass. Balance function was quantitatively evaluated using the Berg Balance Scale (BBS) [29], while muscle mass was analyzed using bioelectrical impedance analysis (BIA) (InBody 370^®^, InBody, Seoul, Republic of Korea) [30]. This method analyzes appendicular skeletal muscle mass (ASM) (kg) and skeletal muscle mass index (SMI) (kg/m^2^) [30]. The ASM was calculated as the sum of the muscle mass of the arms and legs. The SMI was calculated by dividing the ASM value by the square of the height. All outcome measures were evaluated twice, once before the intervention and again three months after the intervention.

Fundamental clinical characteristics, including demographic, radiological, and functional factors, were also investigated.

### 2.6. Statistics

In this study, the sample size was calculated using MET, and the proposed minimum clinically important difference was 1 [8,31]. To meet an alpha level of 0.05 with a power of 0.80, a minimum of 14 subjects were required in each group. Considering a dropout rate of 20%, a minimum of 18 subjects were needed in each group. The sample size calculation was performed using G*Power 3.1.9.7.

The Kolmogorov–Smirnov test was utilized to verify the normal distribution of the data. A paired sample t-test was employed to determine if there were significant changes in CRF indicators, BBS, and SMI when compared at the baseline and three months later. The differences in baseline clinical characteristics and outcome measures between the groups were analyzed using an independent t-test for ordinal scales and a chi-square test for nominal scales. A *p*-value of below 0.05 was defined as statistically significant, and all statistical analyses were performed using SPSS Statistics v.29.0 (IBM SPSS Statistics for Windows, IBM Corp., Armonk, NY, USA).

## 3. Results

### 3.1. Demographic and Clinical Characteristics of the Participants

**Figure 2:**
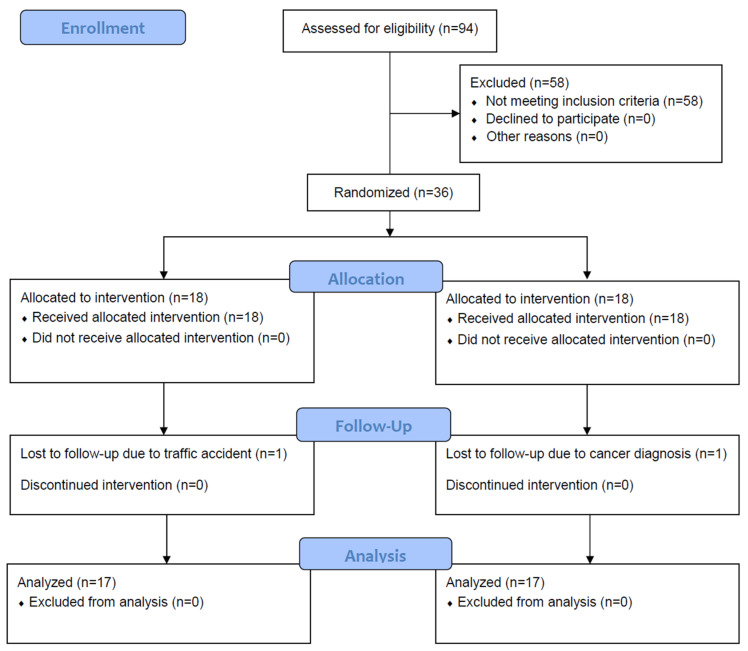
CONSORT flow diagram of recruitment to, allocation within, and participation in this study.

In this study, 94 participants were screened, and 36 patients were deemed suitable for random allocation (Figure 2).

Among the 36 patients, 18 were randomly assigned to the intervention group and 18 to the control group. During the intervention period, one participant from the intervention group and one from the control group were excluded due to a traffic accident and the occurrence of cancer, respectively. Consequently, a total of 34 patients completed the study, with an average age of 70.7 years. There were no significant differences in clinical characteristics between the groups (Table 1). During the HIIT and MICT sessions, no abnormal symptoms, including headache, dizziness, dyspnea, or syncope, or cardiovascular or cerebrovascular attacks, were reported by any of the participants.

### 3.2. Effects of HIIT and MICT on Cardiorespiratory Fitness

The changes in CRF indicators of the patients following HIIT and MICT over three months were analyzed. No significant differences were observed in the indices comprising aerobic function, cardiovascular response, and ventilatory response between the intervention group and the control group prior to the intervention (Table 2).

Both patients who underwent HIIT and those who received MICT showed significant increases in VO_2peak_, VT, and MET_peak_ before and after the intervention (*p* < 0.05, all). When comparing the groups, the intervention group demonstrated significantly greater improvements in VO_2peak_, VT, and MET_peak_ compared to the control group (*p* = 0.005, *p* = 0.002, and *p* = 0.024).

In terms of cardiovascular response, HR_peak_ and O_2_pulse_peak_ both significantly increased after the intervention in both groups (*p* < 0.05, all); however, no significant differences in the extent of increase were observed between the two groups. No significant differences were observed post-intervention in SBP_rest_, DBP_rest_, SBP_peak_, DBP_peak_, or HR_rest_ in either group.

Regarding ventilatory responses, VE_peak_ significantly increased in both HIIT and MICT patients (*p* = 0.032 and *p* = 0.024), yet there was no difference in the degree of improvement between the groups. No significant changes were observed in Vt_peak_, VE/VCO_2peak_, or RR_peak_ before or after the intervention.

### 3.3. Changes in Balance and Muscle Mass After HIIT and MICT

**Figure 3:**
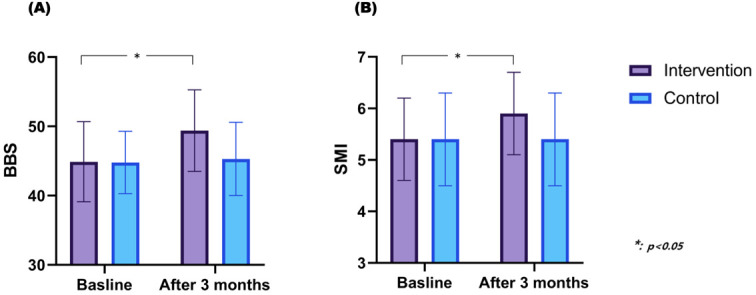
Changes in the BBS and SMI of the participants after 3 months of HIIT and MICT. The intervention group (*n* = 17) that underwent HIIT showed a significant improvement in the BBS and SMI compared to the control group (*n* = 17) that underwent MICT. (**A**) BBS. (**B**) SMI. HIIT, high-intensity interval training; MICT, moderate-intensity continuous training; BBS, Berg Balance Scale; SMI, skeletal muscle mass index.

There was no significant difference in the BBS and SMI between the two groups before the intervention. After the intervention, patients who received HIIT showed a significant increase in the BBS, from 44.9 to 49.4 (*p* = 0.004) (Figure 3A). However, for patients who underwent MICT, no significant change was observed in the BBS, which was 44.8 before the intervention and 45.3 after three months. When comparing the two groups, the degree of improvement in the BBS was significantly different in the intervention group compared to that in the control group (*p* = 0.032).

The SMI in patients who received HIIT significantly increased from 5.4 kg/m^2^ to 5.9 kg/m^2^ after three months (*p* = 0.008) (Figure 3B). However, patients who underwent MICT did not show any significant differences before or after the intervention. Analyzing the degree of improvement between the groups, the intervention group exhibited a significant improvement in the SMI compared to the control group (*p* = 0.032).

## 4. Discussion

The skating exercise, as an HIIT program conducted over three months, was more effective in enhancing CRF in older adults with minor stroke than the MICT program. Additionally, skating exercise demonstrated superior effects in improving balance function and increasing skeletal muscle mass in older adults with minor stroke compared to MICT. It was confirmed that no injuries or adverse effects were observed in all participants who underwent either skating exercise or MICT, indicating that both interventions can be safely implemented for older minor stroke survivors.

Skating exercise as HIIT has demonstrated superior effects in enhancing CRF in elderly patients with minor stroke compared to MICT. Aerobic exercise has been deemed essential in stroke rehabilitation programs, as it aids stroke survivors in improving endurance, walking ability, and overall health [10]. It is known that aerobic exercise is more effective when conducted in conjunction with other exercises such as strength training and balance training [10,32], suggesting that skating exercise may have contributed to a greater enhancement of CRF than MICT. Additionally, previous studies have reported that HIIT is more effective than MICT in improving CRF in stroke patients [14,15,17,18]; however, there are limited HIIT protocols available that are suitable for the elderly population. In particular, elderly stroke patients should consult healthcare professionals regarding the intensity, duration, and type of aerobic exercise to adjust them according to individual needs and capabilities, enabling safe participation in HIIT. In this regard, skating exercise, which allows individuals to adjust the intensity and duration according to their personal needs, offers greater accessibility compared to treadmills for elderly patients.

Generally, it is recommended that healthy adults practice HIIT three times a week for more than 30 min. However, it is challenging for elderly patients with minor stroke to engage in HIIT for 30 min. In previous studies [15], HIIT was performed based on treadmills, and the protocols varied according to patient characteristics. There are many factors to consider, such as the patient’s functional level, age, underlying conditions like heart disease and diabetes, and musculoskeletal pain, which means that the optimized protocol may differ for each patient. Boyne et al. [18] applied HIIT for 25 min once a week three times to chronic stroke patients who had been affected for more than six months. Gjellesvik et al. [17] conducted HIIT on Norwegian stroke patients for 16 min three times a week using a treadmill. Moncion et al. [14] also applied HIIT for 30 min five times a week to stroke patients. The minor stroke patients who participated in this study were on average 70 years old, and the intervention was applied for 20 min, 4 times a week, according to the characteristics of elderly patients.

While improving CRF is important for enhancing post-stroke functionality, managing risk factors, and preventing cardiovascular incidents, preventing injuries during exercise is even more critical. Notably, HIIT constitutes high-intensity aerobic exercise, and elderly patients are at increased risk of injury, even if they have not experienced a stroke [33]. Although skating exercises allow participants to adjust the intensity and duration themselves, they cannot be deemed entirely free from the risk of injury. All participants underwent thorough medical examinations and consultations with rehabilitation medicine physicians to ascertain their fitness for the exercise, and they completed sufficient stretching under the supervision of a physical therapist before performing HIIT and MICT exercises. The physical therapist took measures to report to the doctor immediately when the patient fell during the intervention, but this fall incident during the intervention was not reported.

Elderly patients with minor stroke who underwent skating exercises showed a significantly greater improvement in balance function compared to those who received MICT. Approximately 30% of individuals aged 65 years and older have reported balance dysfunction, and the prevalence continues to rise with age [34]. It has been reported that about 50% of stroke patients experience balance disorders [35], with some studies indicating that up to 80% of elderly stroke patients encounter such issues [36]. Post-stroke balance disorders have a considerable impact on mobility and activities of daily living, increasing the risk of falls [37]. Recent recommendations for balance rehabilitation exercises suggest incorporating various elements, such as proprioceptive sensory training, strength training, agility, plyometrics, and other sport-specific exercises [24]. Skating exercises include most of these components as an effective multilevel intervention, which is hypothesized to enhance balance function compared to steady-state walking or running on a treadmill.

Skating exercises have been shown to aid in increasing overall skeletal muscle mass. Sarcopenia, which is the loss of muscle mass and strength in the elderly population, has been reported to have a prevalence of 10–27% among those aged 60 years and above [38]. Additionally, it is known that 22% of patients with minor stroke experience mild to moderate muscle weakness [39]. Muscle mass indirectly influences the effects of aerobic exercise and is closely related to balance function [30,40]. Skating exercises provide strengthening effects on the quadriceps, gluteus maximus, hamstrings, and calf muscles through their low-intensity squat benefits [24]. Weakness in the lower limb muscles is associated with decreased balance sense; therefore, strength training is an essential component of rehabilitation protocols. Continuous running, which is a key aspect of MICT, also helps strengthen leg muscles and develops fatigue-resistant muscles, which can enhance endurance [41]. While treadmill-based MICT primarily develops slow-twitch muscle fibers that are essential for endurance, skating exercises may be more effective in increasing muscle mass by additionally developing fast-twitch muscle fibers [42].

This study has some limitations. The skater exercise as HIIT effectively improved the aerobic capacity of elderly minor stroke patients compared to MICE, but cardiovascular and ventilatory functions did not improve. The short duration of the intervention may have been a reason for the lack of changes in cardiovascular and ventilatory responses in elderly patients with minor stroke, and pre-existing chronic conditions such as hypertension and diabetes may have had an impact. In addition, participants with minor strokes who may have cognitive impairments that prevent them from understanding the instructions were excluded using the K-MMSE. However, it is possible that the K-MMSE, as a screening test for cognitive impairment, did not completely exclude patients with subtle cognitive impairments. Because this study was conducted with a small number of patients at a single institution, the fact that stratified randomization was not used is another limitation of this research. Bioelectrical impedance analysis has several disadvantages, such as being sensitive to factors like water intake or recent exercise, which can affect accuracy. Lastly, a group that received no intervention was not included in this study. These limitations should be considered when interpreting this study’s results and should be addressed in future research.

## 5. Conclusions

In summary, HIIT was found to be more effective than treadmill-based MICT in improving CRF in elderly patients with minor stroke. It has been confirmed that skating exercises can serve as an HIIT protocol that can be easily and safely utilized by elderly patients with minor stroke. Additionally, skating exercises contributed to enhancing balance function and increasing skeletal muscle mass. In the future, larger-scale multicenter studies should be conducted to clinically utilize skating exercises as a rehabilitation program for elderly patients with minor strokes.

## Figures and Tables

**Table 1:** Demographic and clinical characteristics of the participants.

Factor		Intervention Group (*n* = 17)	Control Group (*n* = 17)	*p*-Value
Age	(years, mean ± SD)	70.5 ± 5.5	70.8 ± 5.7	0.562
Sex	Male	7	7	0.974
	Female	10	10	
Stroke type	Infarct	17	17	0.942
	Hemorrhage	0	0	
Weight	kg	57 ± 8	58 ± 9	0.321
Height	cm	166 ± 9	165 ± 9	0.488
BMI	kg/m^2^	20.7 ± 2.6	21.3 ± 3.1	0.105
Duration after stroke onset	(days)	76.1 ± 8.1	74.1 ± 8.2	0.237
Comorbidity	Hypertension	17	17	0.408
	Diabetes	7	6	
	Hyperlipidemia	15	15	
NIHSS	Onset	1.9 ± 0.8	2.0 ± 0.6	0.207
K-MMSE	(at the beginning of the study)	19.3 ± 4.0	18.5 ± 4.5	0.188

Values are presented as a number (%) or mean ±standard deviation. BMI, body mass index; NIHSS, National Institutes of Health Stroke Scale; and K-MMSE, Korean version of the Mini-Mental State Exam.

**Table 2:** Comparison of changes in cardiorespiratory fitness indicators after 3 months of intervention between the HIIT and MICT group patients.

Cardiorespiratory Fitness	Intervention Group (*n* = 17)		Control Group (*n* = 17)		*p*-Value
	Baseline	After 3 Months	Baseline	After 3 Months	(Cohen’s d)
Aerobic capacities					
VO_2peak_ (mL/kg/min)	20.4 (15.0–25.3)	25.8 (16.5–32.8)	20.6 (14.8–26.1)	23.7 (15.3–29.9)	0.005 * (0.20)
VT (mL/kg/min)	10.6 (4.3–16.4)	15.2 (10.5–19.9)	10.7 (4.5–16.2)	13.1 (5.8–17.3)	0.002 * (0.22)
MET_peak_	6.3 (2.2–10.1)	8.5 (4.3–13.1)	6.5 (2.5–10.5)	7.3 (2.7–11.4)	0.024 * (0.18)
Cardiovascular responses					
SBP_rest_ (mmHg)	125 (101–147)	120 (96–146)	123 (102–146)	119 (100–138)	0.432 (0.12)
DBP_rest_ (mmHg)	76 (60–91)	77 (63–88)	75 (59–90)	78 (60–93)	0.651 (0.14)
SBP_peak_ (mmHg)	171 (122–201)	173 (121–204)	169 (114–200)	174 (117–202)	0.655 (0.12)
DBP_peak_ (mmHg)	77 (57–92)	77 (58–90)	79 (59–93)	80 (60–94)	0.526 (0.18)
HR_rest_ (beats/min)	68 (50–84)	65 (51–80)	66 (49–82)	65 (47–80)	0.871 (0.25)
HR_peak_ (beats/min)	137 (118–156)	140 (122–159)	136 (115–154)	141 (116–153)	0.329 (0.24)
O_2_pulse_peak_ (mL/beat)	8.2 (4.1–11.7)	10.5 (5.3–15.1)	8.0 (4.0–11.1)	10.2 (5.2–14.9)	0.105 (0.24)
Ventilatory responses					
VE_peak_ (L/min)	55 (38–68)	58 (38–66)	56 (39–69)	60 (39–67)	0.210 (0.21)
Vt_peak_ (L)	1.6 (1.3–1.9)	1.6 (1.3–1.9)	1.7 (1.3–2.1)	1.7 (1.3–2.1)	0.488 (0.19)
VE/VCO_2peak_	31.5 (25.4–37.4)	30.9 (25.3–37.2)	30.9 (24.3–37.0)	30.5 (25.0–36.7)	0.429 (0.18)
RR_peak_ (rates/min)	31.2 (23.6–38.5)	32.8 (23.8–38.8)	31.3 (22.5–38.1)	32.6 (23.5–38.1)	0.208 (0.25)

Values are presented as the mean ± standard deviation or number (%). * *p* < 0.05. VO_2peak_, peak oxygen consumption; VT, ventilatory threshold; MET_peak_, peak metabolic equivalents; SBP_rest_, resting systolic blood pressure; DBP_rest_, resting diastolic blood pressure; SBP_peak_, peak systolic blood pressure; DBP_peak_, peak diastolic blood pressure; HR_peak_, resting heart rate; HR_peak_, peak heart rate; O_2_pulse_peak_, peak oxygen pulse; VE_peak_, peak minute ventilation; Vt_peak_, peak tidal volume; VE/VCO_2peak_, peak minute ventilation/carbon dioxide consumption; RR_peak_, peak respiratory rate.

## Data Availability

The original contributions presented in this study are included in the article. Further inquiries can be directed to the corresponding author.
